# Simple new clinical score to predict hepatocellular carcinoma after sustained viral response with direct-acting antivirals

**DOI:** 10.1038/s41598-023-36052-0

**Published:** 2023-06-02

**Authors:** Takao Watanabe, Yoshio Tokumoto, Kouji Joko, Kojiro Michitaka, Norio Horiike, Yoshinori Tanaka, Atsushi Hiraoka, Fujimasa Tada, Hironori Ochi, Yoshiyasu Kisaka, Seiji Nakanishi, Sen Yagi, Kazuhiko Yamauchi, Makoto Higashino, Kana Hirooka, Makoto Morita, Yuki Okazaki, Atsushi Yukimoto, Masashi Hirooka, Masanori Abe, Yoichi Hiasa

**Affiliations:** 1 Department of Gastroenterology and Metabology, Ehime University Graduate School of Medicine, Shitsukawa, Toon, Ehime 791-0295 Japan; 2 Center for Liver-Biliary-Pancreatic Diseases, Matsuyama Red Cross Hospital, 1 Bunkyocho, Matsuyama, Ehime 790-8524 Japan; 3 Department of Gastroenterology, Ehime Prefectural Central Hospital, 83 Kasugamachi, Matsuyama, Ehime 790-0024 Japan; 4 Department of Gastroenterology, Saiseikai Imabari Hospital, 7-1-6 Kitamura, Imabari, Ehime 799-1502 Japan; 5 Department of Gastroenterology, Matsuyama Shimin Hospital, 2-6-5 Ootemachi, Matsuyama, Ehime 790-0067 Japan; 6 Department of Internal Medicine, Saiseikai Matsuyama Hospital, 880-2 Yamanishicho, Matsuyama, Ehime 791-8026 Japan; 7 Department of Gastroenterology, Uwajima City Hospital, 1-1 Gotenmachi, Uwajima, Ehime 798-8510 Japan; 8 Department of Gastroenterology, Ehime Prefectural Imabari Hospital, 4-5-5 Ishiicho, Imabari, Ehime 794-0006 Japan; 9 Department of Gastroenterology, National Hospital Organization Ehime Medical Center, 366 Yokogawara, Toon, Ehime 791-0203 Japan

**Keywords:** Gastrointestinal diseases, Gastrointestinal cancer, Hepatitis

## Abstract

The time point of the most precise predictor of hepatocellular carcinoma (HCC) development after viral eradication with direct-acting antiviral (DAA) therapy is unclear. In this study we developed a scoring system that can accurately predict the occurrence of HCC using data from the optimal time point. A total of 1683 chronic hepatitis C patients without HCC who achieved sustained virological response (SVR) with DAA therapy were split into a training set (999 patients) and a validation set (684 patients). The most accurate predictive scoring system to estimate HCC incidence was developed using each of the factors at baseline, end of treatment, and SVR at 12 weeks (SVR12). Multivariate analysis identified diabetes, the fibrosis-4 (FIB-4) index, and the α-fetoprotein level as independent factors at SVR12 that contributed to HCC development. A prediction model was constructed with these factors that ranged from 0 to 6 points. No HCC was observed in the low-risk group. Five-year cumulative incidence rates of HCC were 1.9% in the intermediate-risk group and 15.3% in the high-risk group. The prediction model at SVR12 most accurately predicted HCC development compared with other time points. This simple scoring system combining factors at SVR12 can accurately evaluate HCC risk after DAA treatment.

## Introduction

Chronic hepatitis C virus (HCV) infection is associated with liver conditions such as cirrhosis, hepatocellular carcinoma (HCC), and liver failure^1–3^. Treatment of HCV-infected patients has advanced with the development of direct-acting antivirals (DAAs), and a sustained virological response (SVR) exceeding 90% has been reported^4–6^. Moreover, 12 weeks of sofosbuvir/velpatasvir therapy for patients with HCV-related decompensated cirrhosis, considered to be Child–Pugh class B or C, and a rate of sustained virological response 12 weeks after end of therapy (SVR12) of 92% have been achieved with good safety^7^.

The ultimate goal of treating chronic HCV infection is to prevent liver-related death, including HCC. In several past studies, treatment with DAAs reduced the risk of HCC and liver disease-related death^8,9^. A meta-analysis by Waziry et al. concluded that, after adjusting for age, observation periods, and interferon (IFN)/ DAA therapy did not alter the incidence of HCC after viral clearance^10^.

The incidence of HCC after SVR has been reported to be 0.38–1.82/100 patient-years for IFN therapy and 0.9–2.96/100 patient-years for DAA therapy^10–13^. Even after viral eradication with DAA therapy, HCC can sometimes occur. Therefore, patients who achieve SVR should be closely monitored for the development of HCC. Because the determination of HCC risk strongly affects subsequent surveillance, several studies have reported risk factors for the development of HCC and characteristics of patients who develop HCC after SVR with DAA^11^. As with IFN therapy, older age, male sex, liver fibrosis, and posttreatment alpha-fetoprotein (AFP) levels are factors related to HCC development after DAA therapy^14–16^; dysplastic nodules^17^ and posttreatment M2BPGi^18^ have also been reported as factors.

However, after HCV eradication, significant changes in laboratory values are expected due to improvement in liver function and regression of liver fibrosis^19^. In this context, the cut-off value at which HCC development can be predicted depends on the time of evaluation, but it is not yet clear which time point after viral eradication with DAA most accurately predicts HCC incidence.

Furthermore, the modality and frequency of testing for HCC screening should be adjusted according to the individual risk of HCC incidence, while taking cost-effectiveness into account. In other words, a simple HCC risk assessment system would provide personalized follow-up system for patients with achievement of SVR.

The purpose of this study was to develop a scoring system that can predict HCC occurrence after DAA treatment by comparing prediction models at baseline, at the end of treatment (EOT), and at SVR12, respectively, and then use it to adopt appropriate surveillance according to the risk of HCC occurrence.

## Results

### Study cohort characteristics

Table 1 summarizes the characteristics of the training and validation sets. In the training set, the patients included 448 males (44.8%), with age of 65.6 ± 10.3 years at baseline. At baseline, platelet count was 16.4 ± 6.6 × 10^4^/µL, FIB-4 index was 3.6 ± 3.7, serum albumin level was 4.1 ± 0.4 g/dL, and AFP was 10.1 ± 25.3 ng/mL. Diabetes mellitus (DM) was present in 170 patients (17.0%). The HbA1c levels of patients without and with DM were 5.5 ± 0.3% and 7.1 ± 1.2% respectively. Table 1 also shows the clinical factors at EOT and SVR12. There were no differences in the laboratory data between the two cohorts.

**Table 1:** Clinical and virological characteristics of patients in the training and validation sets at baseline and after DAA therapy.

	Training set (n = 999)	Validation set (n = 684)	*p*-value
Data at baseline			
Age (y)	65.6 ± 10.3	66.0 ± 10.2	0.36
Male (n, %)	448, 44.8	318, 46.4	0.51
Body mass index (kg/m^2^)	23.2 ± 3.8	23.1 ± 4.0	0.41
White blood cell count (/µL)	5076 ± 1634	4914 ± 1498	0.037
Platelet count (× 10^4^/µL)	16.4 ± 6.6	16.1 ± 7.0	0.35
ALT (U/L)	51.0 ± 45.7	49.7 ± 45.6	0.54
AST (U/L)	48.9 ± 34.0	48.5 ± 33.3	0.81
Total bilirubin (mg/dL)	0.8 ± 0.6	0.7 ± 0.3	0.54
Albumin (g/dL)	4.1 ± 0.4	4.1 ± 0.4	0.53
Prothrombin time (%)	93.2 ± 17.0	93.4 ± 16.3	0.82
AFP (ng/mL)	10.1 ± 25.3	8.8 ± 16.3	0.13
Diabetes mellitus (n, %)	170, 17.0	106, 15.5	0.42
LDL cholesterol (mg/dL)	96.7 ± 29.0	94.9 ± 31.2	0.39
Triglycerides (mg/dL)	117 ± 75	108 ± 57	0.074
Alcohol (none/drinker/unknown)	648/130/221	449/83/152	0.78
Smoking (n, %)	221, 22.1	149, 21.7	0.90
FIB-4 index	3.6 ± 3.7	3.6 ± 2.6	0.94
HCV genotype (1/2)	687/308	450/227	0.28
HCV RNA (log copies/mL)	5.9 ± 0.8	5.9 ± 0.8	0.83
HBV coinfection (n, %)	12, 1.2	5, 0.7	0.45
Data at EOT			
White blood cell count (/µL)	5223 ± 1760	5080 ± 1752	0.12
Platelet count (× 10^4^/µL)	17.0 ± 6.9	16.7 ± 7.1	0.36
ALT (U/L)	20.5 ± 15.8	21.0 ± 15.0	0.54
AST (U/L)	25.0 ± 12.7	25.6 ± 12.5	0.35
Total bilirubin (mg/dL)	0.8 ± 0.4	0.8 ± 0.4	0.87
Albumin (g/dL)	4.1 ± 0.3	4.1 ± 0.3	0.34
Prothrombin time (%)	94.8 ± 17.3	93.7 ± 17.4	0.23
AFP (ng/mL)	4.3 ± 3.3	4.6 ± 3.8	0.33
FIB-4 index	2.8 ± 2.8	2.9 ± 2.0	0.83
Data at SVR12			
White blood cell count (/µL)	5513 ± 1777	5366 ± 1622	0.11
Platelet count (× 10^4^/µL)	16.8 ± 6.7	16.2 ± 5.9	0.11
ALT (U/L)	18.5 ± 10.9	21.0 ± 36.8	0.11
AST (U/L)	24.2 ± 9.5	27.1 ± 34.6	0.046
Total bilirubin (mg/dL)	0.7 ± 0.3	0.8 ± 0.4	0.13
Albumin (g/dL)	4.2 ± 0.3	4.2 ± 0.3	0.62
Prothrombin time (%)	93.5 ± 16.1	93.4 ± 15.5	0.91
AFP (ng/mL)	4.0 ± 3.4	4.1 ± 3.2	0.97
FIB-4 index	2.8 ± 2.3	2.9 ± 2.0	0.16
DAA regimen			
ASV/DCV	95	79	
SOF/LDV	387	239	
SOF/RBV	215	162	
OBV/PTV/r	74	37	
EBR/GZR	47	36	
GLE/PIB	174	121	
SOF/VEL	7	10	

Data are expressed as means ± standard deviation.

DAA, direct-acting antiviral; ALT, alanine aminotransferase; AST, aspartate aminotransferase; AFP, α-fetoprotein; LDL, low-density lipoprotein; FIB-4, fibrosis-4; HCV, hepatitis C virus; RNA, ribonucleic acid; HBV, hepatitis B virus; EOT, end of treatment; SVR, sustained virological response; ASV, asunaprevir; DCV, daclatasvir; SOF, sofosbuvir; LDV, ledipasvir; RBV, ribavirin; OBV, ombitasvir; PTV, paritaprevir; r, ritonavir; EBR, elbasvir; GZR, grazoprevir; GLE, glecaprevir; PIB, pibrentasvir; VEL, velpatasvir.

### Change in fibrosis, liver function, and AFP

The FIB-4 index as a surrogate marker of liver fibrosis, serum albumin level, PT (%) as a marker of liver function, and the AFP level were measured at baseline, EOT, and SVR12 in all analyzed patients (Fig. S1).

The FIB-4 index was 3.62 ± 3.36, 2.88 ± 2.55, and 2.90 ± 2.26 at baseline, EOT, and SVR12, respectively. The FIB-4 index at EOT and SVR12 was significantly decreased compared with baseline (Fig. S1a). The mean serum albumin level was 4.1 ± 0.4, 4.1 ± 0.3, and 4.2 ± 0.3 g/dL, respectively. The serum albumin level was not changed at EOT; however, the level at SVR12 was significantly higher than at EOT (Fig. S1b). PT activity (%) was 93.4 ± 16.5%, 94.3 ± 17.4%, and 93.5 ± 15.8%, respectively. PT activity at EOT and SVR12 was not significantly different compared with baseline (Fig. S1c). The serum AFP level was 9.6 ± 22.1, 4.5 ± 3.7, and 4.1 ± 3.5 ng/mL, respectively. The serum AFP level at EOT and SVR12 was significantly lower than at baseline (Fig. S1d).

### Cumulative HCC incidence

**Figure 1:**
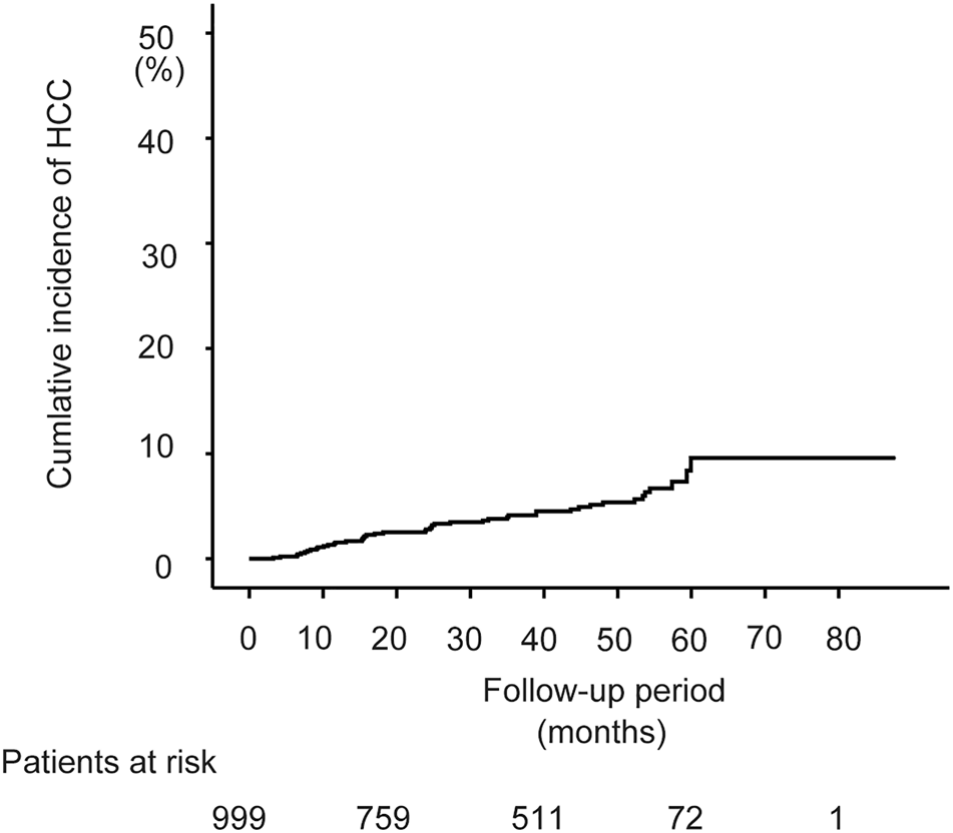
Kaplan–Meier curves of the cumulative incidence of hepatocellular carcinoma (HCC) after the end of DAA therapy in the training set.

In the training set, the median observation period was 1,121 days (38 ± 18 months) after EOT. HCC developed in 46 cases (5.4%) during the study period. The overall cumulative incidence of HCC was 1.5%, 4.1%, and 9.6% at 1, 3, and 5 years after EOT, respectively (Fig. 1). In the validation set, HCC developed in 37 cases (5.4%) during the study period. The overall cumulative incidence of HCC was 2.0%, 4.2%, and 8.2% at 1, 3, and 5 years after EOT, respectively. The cumulative HCC incidence rates were not different between the training and validation sets (log rank test, *p* = 0.39).

### Predictors for development of HCC after achievement of SVR with DAA therapy

Factors that may be associated with the development of HCC after DAA treatment were examined at each time point in the training set.

At baseline, univariate analysis identified age (*p* = 0.029), sex (*p* = 0.001), DM (*p* = 0.001), WBC (*p* = 0.009), platelet count (*p* < 0.001), albumin level (*p* < 0.001), PT (*p* < 0.001), and the FIB-4 index (*p* < 0.001) as predictors (Table S1). Multivariate analysis identified male sex (HR = 2.08; 95% CI 1.06–4.04, *p* = 0.031), diabetes mellitus (HR = 2.51; 95% CI 1.27–4.95, *p* = 0.008), white blood cell count at baseline (HR = 0.99; 95% CI 0.99–0.99, *p* = 0.001), and prothrombin activity at baseline (HR = 0.97; 95% CI 0.96–0.99, *p* = 0.002) as independent factors that contributed to the development of HCC (Table S1) Age and platelet count were excluded in the multivariate analysis because they are confounding factors of the FIB-4 index.

At EOT, univariate analysis identified age (*p* = 0.029), sex (*p* = 0.001), DM (*p* = 0.001), the same as in Table S1 and platelet count (*p* < 0.001), ALT (*p* = 0.002), AST (*p* < 0.001), albumin level (*p* = 0.024), PT (*p* < 0.001), AFP (*p* < 0.001), and the FIB-4 index (*p* < 0.001) as predictors (Table S2). Multivariate analysis identified male sex (HR = 4.84; 95% CI 1.83–12.8, *p* = 0.001), FIB-4 index at EOT (HR = 1.09; 95% CI 1.01–1.18, *p* = 0.028), and the AFP level at EOT (HR = 1.13; 95% CI 1.06–1.19, *p* < 0.001) as independent factors that contributed to the development of HCC (Table S2). Age, platelet, ALT, and AST were excluded in the multivariate analysis because they are confounding factors of the FIB-4 index.

At SVR12, univariate analysis identified age (*p* = 0.029), sex (*p* = 0.001), DM (*p* = 0.001), the same as in Table S1, and platelet count (*p* = 0.006), AST (*p* = 0.017), PT (*p* = 0.002), AFP (*p* = 0.004), and the FIB-4 index (*p* < 0.001) at SVR12 as predictors (Table 2). Multivariate analysis identified DM (HR = 3.95; 95% CI 1.19–10.7, *p* = 0.023), FIB-4 index at SVR12 (HR = 1.11; 95% CI 1.04–1.18, *p* < 0.001), and the AFP level (HR = 1.05; 95% CI 1.00–1.11, *p* = 0.035) at SVR12 as independent factors that contributed to the development of HCC (Table 2). Age, platelet, and AST were excluded in the multivariate analysis because they are confounding factors of the FIB-4 index.

**Table 2:** Factors 12 weeks after the end of treatment associated with the occurrence of HCC after DAA treatment in the training set.

	HCC occurrence		Univariate analysis		Multivariate analysis	
	Yes	No	Hazard ratio (95% CI)	*p*-value	Hazard ratio (95% CI)	*p*-value
Age (y)	68.9 ± 8.2	65.4 ± 10.3	1.03 (1.00–1.06)	0.029		
Male (n, %)	31, 67.3	416, 43.7	2.75 (1.48–5.10)	0.001	–	–
Body mass index (kg/m^2^)	24.1 ± 3.1	23.2 ± 3.8	1.06 (0.98–1.14)	0.106		
Diabetes mellitus (n, %)	16, 34.7	154, 16.2	2.73 (1.49–5.02)	0.001	3.59 (1.19–10.7)	0.023
Alcohol (n, %)	5, 16.6	125, 16.7	1.17 (0.63–2.18)	0.60		
Smoking (n, %)	14, 6.3	32, 4.1	1.60 (0.85–3.00)	0.14		
HBV coinfection (n, %)	1, 8.3	45, 4.5	1.65 (0.22–12.0)	0.61		
White blood cell count (/µL)	4922 ± 1786	5545 ± 1774	1.00 (0.99–1.00)	0.072		
Platelet count (× 10^4^/µL)	14.1 ± 8.5	16.9 ± 6.6	0.92 (0.87–0.97)	0.006		
ALT (U/L)	21.0 ± 10.9	18.4 ± 10.8	1.01 (0.99–1.04)	0.108		
AST (U/L)	27.4 ± 7.5	24.0 ± 9.5	1.02 (1.00–1.05)	0.017		
Total bilirubin (mg/dL)	0.8 ± 0.3	0.7 ± 0.3	1.38 (0.70–2.74)	0.34		
Albumin level (g/dL)	4.1 ± 0.3	4.2 ± 0.3	0.56 (0.26–1.24)	0.15		
Prothrombin activity (%)	85.6 ± 15.9	93.9 ± 16.0	0.97 (0.95–0.99)	0.002	-	-
AFP level (ng/mL)	6.8 ± 4.1	4.0 ± 3.3	1.06 (1.02–1.19)	0.004	1.05 (1.00–1.11)	0.035
FIB-4 index	4.5 ± 5.2	2.7 ± 2.0	1.09 (1.05–1.14)	< 0.001	1.11 (1.04–1.18)	< 0.001

Data are expressed as means ± standard deviation.

HCC, hepatocellular carcinoma; DAA, direct-acting antiviral; CI, confidence interval; ALT, alanine aminotransferase; AST, aspartate aminotransferase; AFP, α-fetoprotein; FIB-4, fibrosis-4; SVR, sustained virological response.

### Cumulative HCC incidence rate stratified by risk factors

First, the cumulative HCC incidence rate was examined with the factors extracted in the baseline data. Males had a significantly higher cumulative incidence rate than females (log-rank test, *p* < 0.001, Fig. S2a). In patients with DM, cumulative occurrence was significantly higher than in those without DM (*p* < 0.001, Fig. S2b). There were significant differences in HCC occurrence when patients were divided into three groups by the first and second tertiles of the white blood cell count and the prothrombin activity level (*p* = 0.045, and 0.003, respectively) (Fig. S2c, S2d). The first/second tertiles of the white blood cell count and the prothrombin activity level at baseline were 4223/5600/µL, and 87.1/99.7%, respectively.

AT EOT, there were significant differences in the development of HCC when patients were divided into three groups by the first and second tertiles of the FIB-4 index and the AFP level (*p* < 0.001, and 0.033, respectively) (Figs S3a, S3b). The first/second tertiles of the FIB-4 index and AFP were 1.7/2.8, and 3.0/4.6 ng/mL, respectively.

**Figure 2:**
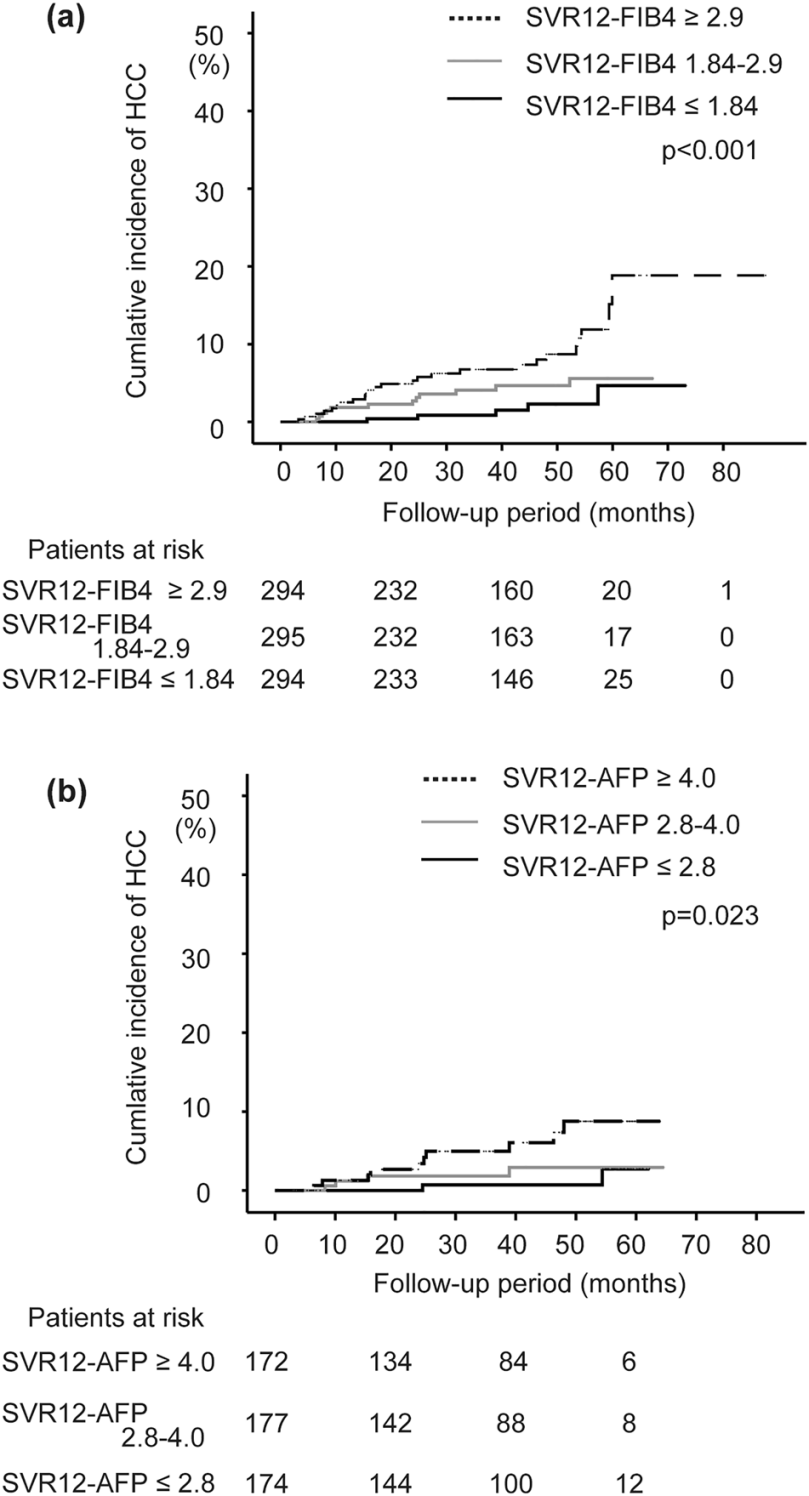
Comparison of cumulative HCC incidence in the training set. There is a significant difference among the patients by FIB-4 index at SVR12 (*p* < 0.001) (**a**) and AFP level (ng/mL) at SVR12 (*p* = 0.023) (**b**) with the log-rank test.

When the three groups were divided by the first and second tertiles of the FIB-4 index and of the AFP level at SVR12, there were significant differences in the development of HCC (*p* < 0.001, and 0.023, respectively) (Fig. 2a,b). The first/second tertiles of the FIB-4 index and the AFP level at SVR12 were 1.84/2.90, and 2.8/4.0 ng/mL, respectively.

### Scoring system to evaluate the risk of HCC development after SVR by DAA treatment

Based on the results of the multivariate analysis with factors at baseline, a scoring system was constructed at each time point (Table 3). Namely, male and cases with DM were scored as 2 points, and other continuous variables were scored as 0 to 2 points based on their first and second tertiles.

**Table 3:** Scoring system for prediction of HCC occurrence.

Factor	Score		
	0	1	2
a) Data at baseline (0–8 points)			
Sex	Female	–	Male
Diabetes mellitus	no	–	Yes
White blood cell count	≥ 5600	4223–5600	≤ 4223
Prothrombin activity	≥ 99.7	87.1–99.7	≤ 87.1
b) Data at end of treatment (EOT) (0–6 points)			
Sex	Female	–	Male
FIB-4 index	≤ 1.7	1.7–2.8	≥ 2.8
AFP level	≤ 3.0	3.0–4.6	≥ 4.6
c) Data at sustained virological response 12 weeks after end of therapy (SVR12) (0–6 points)			
Diabetes mellitus	No	–	Yes
FIB-4 index	≤ 1.84	1.84–2.90	≥ 2.90
AFP level	≤ 2.8	2.8–4.0	≥ 4.0

HCC, hepatocellular carcinoma; AFP, α-fetoprotein; FIB-4, fibrosis-4.

**Figure 3:**
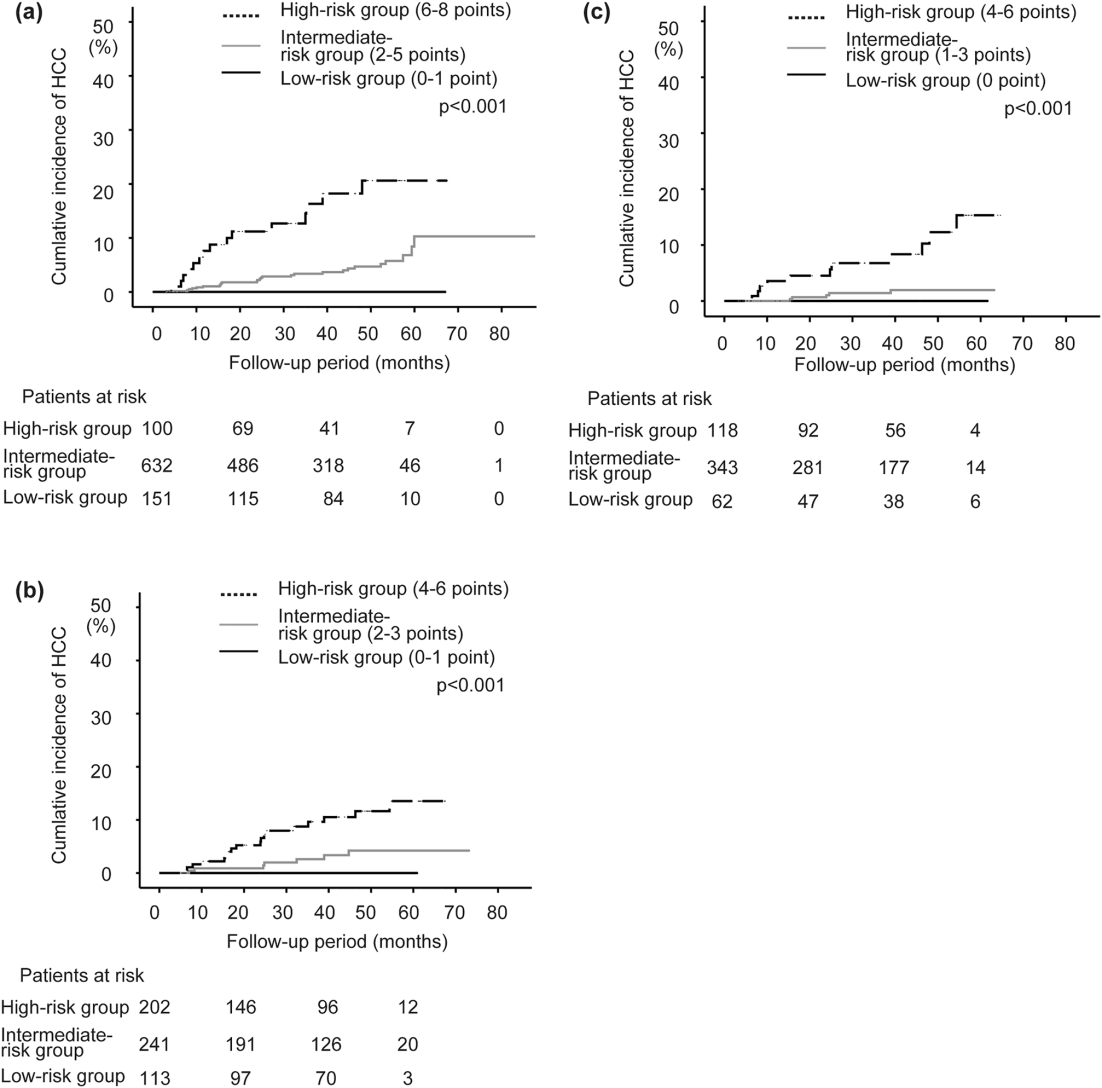
Comparison of cumulative HCC incidence in the training set by the scoring systems with factors at each time point. The cumulative occurrence of HCC by group is significantly stratified by the score at baseline (*p* < 0.001) (**a**), EOT (*p* < 0.001) (**b**), and SVR12 (*p* < 0.001) (**c**) with the log-rank test.

At baseline, the patients were then grouped based on these scores as follows: 0–1 point, low-risk group (n = 151); 2–5 points, intermediate-risk group (n = 632); and 6–8 points, high-risk group (n = 100). Figure 3a shows the cumulative incidence of HCC for each group. The respective 2-, 3-, 4-, and 5-year cumulative incidence rates of HCC were 0, 0, 0, and 0% in the low-risk group; 2.2, 3.4, 4.7, and 10.3% in the intermediate-risk group; and 11.2, 16.3, 20.6, and 20.6% in the high-risk group. The cumulative HCC incidence was stratified significantly (*p* < 0.001).

At EOT, grouping was performed using these scores as follows: 0–1 point, low-risk group (n = 113); 2–3 points, intermediate-risk group (n = 241); and 4–6 points, high-risk group (n = 202). Figure 3b shows the cumulative incidence of HCC for each group. The respective 2-, 3-, 4-, and 5-year cumulative incidence rates of HCC were: 0, 0, 0, and 0% in the low-risk group; 0.9, 2.6, 4.2, and 4.2% in the intermediate-risk group; and 6.6, 9.6, 11.6, and 13.5% in the high-risk group. The cumulative HCC incidence was stratified significantly (*p* < 0.001).

Finally, a scoring system was constructed (score ranged from 0 to 6 points) at SVR12 as follows: 0 points, low-risk group (n = 62); 1–3 points, intermediate-risk group (n = 343); and 4–6 points, high-risk group (n = 118). Figure 3c shows the cumulative incidence of HCC for each group. No HCC occurred in the low-risk group. The respective 2-, 3-, 4-, and 5-year cumulative incidence rates of HCC were 1.0, 1.4, 1.9, and 1.9%, respectively, in the intermediate-risk group and 4.5, 6.8, 12.3, and 15.3%, respectively, in the high-risk group. The cumulative HCC incidence was stratified significantly (*p* < 0.001).

### Comparison of prediction accuracy for HCC occurrence

**Figure 4:**
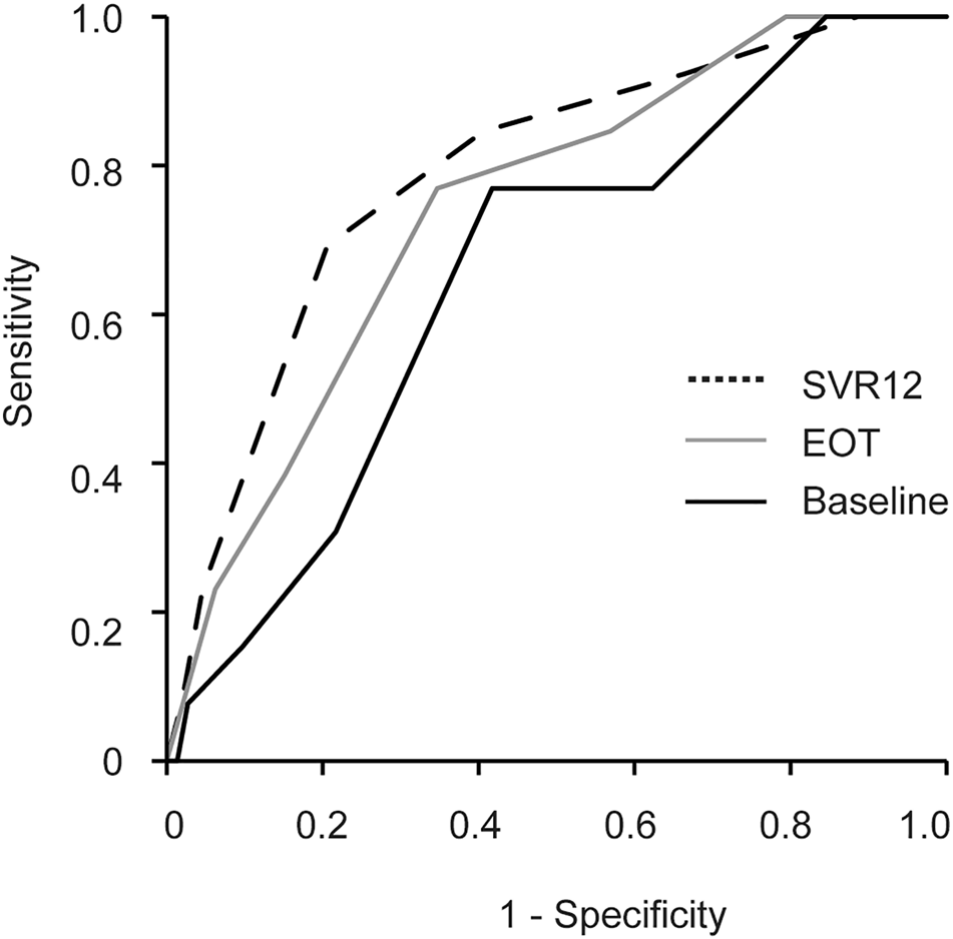
Receiver-operating characteristic (ROC) curves in the training set for the score derived from data at baseline (black line), EOT (gray line), and SVR12 (dotted line). The area under the curve (AUC) value for the score at baseline, EOT, and SVR12 is 0.654, 0.740, and 0.793, respectively.

Since all three scoring systems were able to stratify the occurrence of HCC significantly, it was decided to examine which model was the most efficient. Prediction accuracy for HCC development after SVR with DAA treatment was compared by ROC analysis among the scores at baseline, EOT, and SVR12 (Fig. 4). It was found that scoring at SVR12 had higher AUC (AUC, 0.793; 95%CI, 0.691–0.894; *p* < 0.001) than at baseline (AUC, 0.654; 95%CI, 0.517–0.79; *p* = 0.027) and at EOT (AUC, 0.74; 95%CI, 0.616–0.865; *p* < 0.001). When the optimal cut-off value of the score at SVR12 was set as 3 (as obtained from the training set), the sensitivity and negative predictive value (NPV) were 87.5% and 59.8%, respectively.

### HCC incidence excluding patients who developed HCC within a shorter period

Considering the possibility that HCC that could not be detected by imaging studies was already present at the end of DAA treatment, the scoring system was applied at SVR12 excluding patients who developed HCC within 6, 9, and 12 months after the end of DAA treatment respectively. The cumulative HCC incidence could also be significantly stratified with exclusion of patients whose observation period was within 6, 9, and 12 months (Fig S4a–c; *p* < 0.001 at 6 months; *p* = 0.002 at 9 months; *p* = 0.006 at 12 months).

### Validation of the prediction model

**Figure 5:**
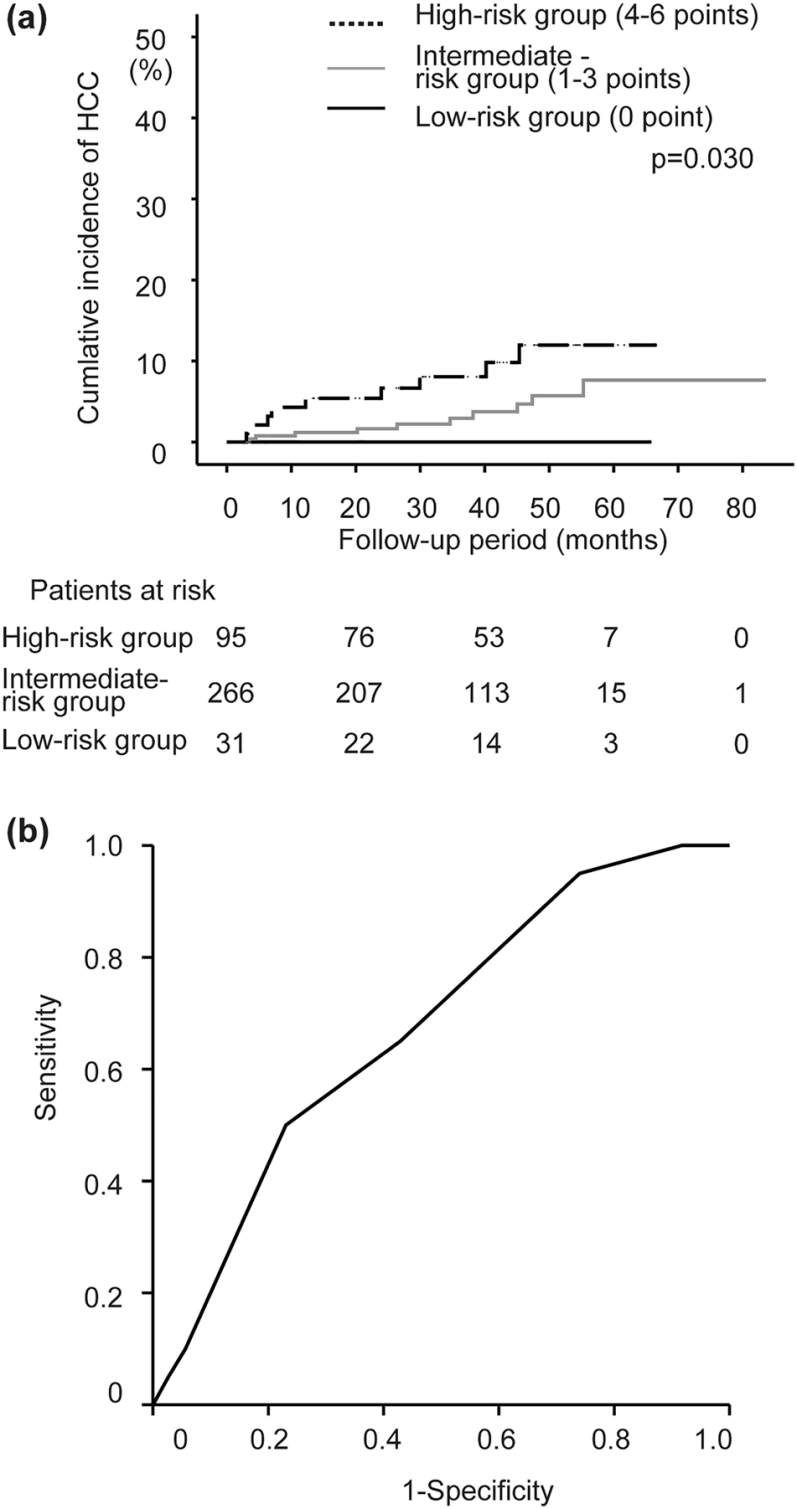
The cumulative incidence of HCC in the validation set by stratifying the patients into three groups according to the score (0–6 points): low-risk group (0 points), intermediate-risk group (1–3 points), and high-risk group (4–6 points). The total score was obtained by summing diabetic mellitus (yes: 2, no: 0), FIB-4 index at SVR12 (0: ≤ 1.84, 1: 1.84–2.90, 2: ≥ 2.90), and AFP level (0: ≤ 2.8, 1: 2.8–4.0, 2: ≥ 4.0 ng/mL) at SVR12. The cumulative HCC incidence increases significantly with higher scores determined in the training set by the log-rank test (*p* = 0.030) (**a**). Receiver-operating characteristic (ROC) curves in the validation set for the score derived from data at SVR12. The area under the curve (AUC) value for the score at SVR12 in the validation set is 0.674 (**b**).

To validate the prediction model for HCC occurrence after SVR, the cumulative HCC incidence rates were compared among patients grouped based on scores at SVR12, shown in Table 3 in the validation set. The study patients of the validation set were grouped based on the scoring of the training set at SVR12 as follows: low-risk group (n = 31), intermediate-risk group (n = 266), and high-risk group (n = 95). Figure 5a shows the cumulative incidence of HCC. The respective 2-, 3-, 4-, and 5-year cumulative occurrence rates of HCC were: 0, 0, 0, and 0% in the low-risk group; 1.6, 3.3, 6.9, and 9.6% in the intermediate-risk group; and 6.7, 8.1, 12.0, and 12.0% in the high-risk group. The cumulative HCC incidence was stratified significantly (*p* = 0.03). Prediction accuracy for HCC development after SVR with DAA treatment in the validation set was confirmed by ROC analysis (Fig. 5b). It was found that scores at SVR12 could significantly predict HCC development (AUC, 0.674; 95%CI, 0.563–0.784; *p* = 0.009). When the optimal cut-off value of the score was set as 3 (as obtained from the training set), the sensitivity and NPV were 65.0% and 57.1%, respectively.

## Discussion

Which time point is the most accurate to determine the risk of HCC developing after SVR within the EKEN study was examined. At SVR12, the scores calculated by the presence of DM and the FIB-4 and AFP values showed the highest accuracy.

We previously reported predictors of HCC after DAA therapy^14^. In this report, multivariate analysis identified the FIB-4 index and AFP at EOT as independent factors that contributed to the development of HCC after DAA therapy within the median observation period of 537 days. Improvement in liver function and regression of liver fibrosis are expected after HCV eradication^19^. Liver stiffness decreases rapidly after DAA therapy, even in cirrhotic patients, suggesting resolution of inflammation^20^. Similarly, pre-treatment clinical factors reflect the influence of inflammation or the immune response on HCV infection^18,21,22^. In fact, FIB-4 and AFP decreased and albumin and PT improved from baseline to EOT and SVR12; thus, achievement of SVR may have a strong impact on the development of HCC by improving liver fibrosis and inflammation. Despite several proposed risk factors for HCC after SVR, it has not yet been fully determined at which point it is appropriate to assess the risk of HCC occurrence. Therefore, the present study examined which of the time points, i.e., baseline, EOT, and SVR12, showed the best performance for HCC prediction, and it was found that scores at SVR12 most accurately predicted HCC development compared to scores at baseline and EOT.

In a real-world, multicenter study in Japan, in patients with compensated cirrhosis at SVR12, the serum albumin level improved 18% at EOT and 27% at SVR12^23^. In patients with decompensated cirrhosis at SVR12, similar improvement in the albumin level was seen. The current data showed that albumin was not significantly different from baseline to EOT, but it was significantly increased at SVR12. Rout et al. evaluated changes in liver stiffness measurement with transient elastography after DAA therapy. Liver stiffness decreased from 6.9 (5.1–12.7) kPa at baseline to 6.1 (4.8–9.4) kPa 1 year after SVR^24^. Liver fibrosis was evaluated with the FIB-4 index as a non-invasive fibrosis test, and baseline, EOT, and SVR12 values were compared. It was found that the FIB-4 index was significantly decreased at SVR12 compared to baseline. To evaluate the risk of developing HCC accurately and conveniently after SVR in the present study, it was considered that evaluating the data at SVR12, which is after improvement in liver function and hepatic fibrosis due to virus elimination, was more useful than evaluating the data at DAA treatment initiation or EOT.

A recent study using multivariate Cox proportional hazards analysis showed that the baseline body mass index (≥ 25.0 kg/m^2^), baseline FIB-4 index (≥ 3.25), albumin level at SVR (< 4.0 g/dL), and AFP at SVR (≥ 5.0 ng/mL) were significantly associated with the development of HCC^25^. This group used these four parameters and developed a model to predict the development of HCC^25^. In this report, the FIB-4 index was based on the value at baseline, and a cut-off value of 3.25 was used, which is a well-known predictive value for advanced fibrosis^26^. In the present study, the FIB-4 index at SVR12 was used, and the cut-off value selected was much lower than 3.25 because the FIB-4 index decreased at SVR12 compared to baseline. Because this reduction may be affected by changes in inflammation and the immune response due to HCV eradication, the FIB-4 index at SVR12 may be better for assessing the risk of HCC after SVR^27^. Because the FIB-4 index is expected to decrease after SVR, HCC risk assessment should carefully consider the time point of assessment.

A higher AFP value was reported to be associated with the risk for the development of HCC after SVR with IFN-based therapy^21,22^. Similarly, several reports with DAA therapy identified the AFP level as an independent factor related to the incidence of HCC after SVR^25,28^. The cut-off value of AFP at EOT was reported as 9.0 ng/mL^28^. The oncogenic potential of HCV-infected hepatocytes is thought to remain at EOT, and it gradually decreases with HCV eradication. In the present study, the cut-off value was 4.0 ng/mL. Similarly, the cut-off value of AFP at SVR12 was reported to be 5.0 ng/mL^25^. These results might mean that lower cut-off value of AFP is needed to appropriately evaluate the potential of HCC incidence after DAA therapy.

In the present study, DM was an independent risk factor for the development of HCC after SVR with DAA therapy. Type 2 DM causes a 1.7-fold increased risk for the development of HCC in HCV-positive patients treated with IFN^29^. In a systematic review and meta-analysis, DM was a risk factor for the development of HCC after SVR with DAA treatment^30^. Regarding medication for DM, the use of metformin in patients with DM may reduce the risk of HCC^31,32^. In our present study, 19 patients were taking metformin at the start of DAA therapy; HCC developed in 0 of 19 patients who were taking metformin and in 16 of 135 patients who were not taking metformin. It is possible that the development of HCC is suppressed in patients taking metformin, but the number of patients taking metformin was small, so the results showed no significant difference (*p* = 0.12). One of the limitations of this study is the lack of detailed data about the treatment and the clinical course of DM before or after the start of DAA treatment.

In the present study, factors including known parameters such as DM, FIB-4, and AFP were identified. However, previous reports did not examine the optimal time point for accurate prediction of HCC occurrence after SVR, even though the values of FIB-4 and AFP change with DAA treatment. Therefore, the risk factors were compared using data at baseline, EOT, and SVR12. The model using the data at SVR12 was the most accurate for predicting the development of HCC. Finally, a simple and accurate scoring system to assess the risk of HCC using precise cut-off values at SVR12 was proposed.

The present study had some limitations. The presence of dysplastic nodules, which show hypointense lesions on the hepatobiliary phase on Gd-EOB-MRI, was reported to be useful in predicting the development of HCC after SVR^17,33^. However, most of the cases underwent dynamic CT, and few cases underwent Gd-EOB-MRI before treatment in the present study. Therefore, the usefulness of Gd-EOB-MRI could not be evaluated. In addition, the cost of Gd-EOB-MRI is high, and it is difficult to implement Gd-EOB-MRI in all patients before DAA treatment, which includes those with a low probability of developing HCC, considering cost-effectiveness. Therefore, the present model, which does not require special testing, appears to be easier to use in general practice.

In this report, a new scoring system that combines three factors at SVR12 with DAA treatment and accurately evaluates the risk of HCC was proposed. This scoring system allowed stratification of patients according to their risk of developing HCC. The 5-year cumulative incidence rate of HCC was 0% (low-risk group) to 15.3% (high-risk group). More frequent examinations using precise imaging modalities such as dynamic CT or MRI may be recommended in high-risk patients. On the other hand, relatively simple tests such as ultrasonography and/or tumor marker measurement may be sufficient in low-risk patients. This scoring system may be very useful in terms of cost–benefit performance after achieving SVR.

## Methods

### Patients

A multicenter study was performed involving 10 hospitals, Ehime Kan-En Network (EKEN; Ehime University Hospital, Matsuyama Red Cross Hospital, Ehime Prefectural Central Hospital, Uwajima City Hospital, Saiseikai Imabari Hospital, Matsuyama Shimin Hospital, Ehime Prefectural Imabari Hospital, Ehime Prefectural Niihama Hospital, Saiseikai Matsuyama Hospital, and National Hospital Organization Ehime Medical Center). Between 2014 and 2020, 1,793 consecutive patients with chronic HCV infection without a history of HCC were treated with DAAs without IFN. Patients who had a history of HCC or who did not achieve SVR and whose observation periods were shorter than 12 weeks after the end of DAA therapy were excluded. A total of 1,683 patients were finally analyzed in the present study. A retrospective medical chart review of these patients was performed, none of whom had a previous history of HCC, as determined by helical dynamic computed tomography (CT) or Gd-EOB-magnetic resonance imaging (MRI).

Patients who were treated with warfarin at the time of initiation of DAA therapy were excluded from prothrombin activity (PT) analysis. Diabetes mellitus (DM) was defined as HbA1c > 6.5% or treatment with anti-diabetes drugs or insulin prior to DAA treatment. An alcohol drinker was defined as a patient who consumed 20 g or more of alcohol per day.

The Ethics Committee of Ehime University Hospital (Approval ID 1,411,010) approved this study, which was performed according to the ethical guidelines of the Declaration of Helsinki amended in 2008. All patients provided written, informed consent prior to inclusion in this study.

### Clinical and laboratory data collection

Clinical and laboratory data were collected at baseline, end of treatment (EOT), and SVR12. The Roche COBAS^®^ TaqMan® HCV Auto assay system (Roche Molecular Diagnostics, Pleasanton, CA) was used to measure HCV RNA levels. This system has a lower limit of quantification of 1.2 log_10_ IU/mL. The fibrosis-4 (FIB-4) index was calculated and used as a surrogate indicator of liver fibrosis^26^.

### Surveillance for HCC

Following completion of DAA therapy, patients were assessed every 3–6 months for biochemical and virological values and blood counts. In addition, ultrasonography, helical dynamic CT, or MRI were used to screen for HCC. HCC was diagnosed according to the presence of typical hypervascular characteristics on angiography or findings on dynamic CT or MRI. Fine-needle aspiration biopsy and histological examination were performed to diagnose HCC in the absence of typical HCC findings.

### Statistical analysis

The 1683 patients were split into two cohorts: a training set and a validation set. Patients in the validation set were selected at a ratio of 3:2 based on patients in the training set by using the random function in SPSS software ver. 23 (IBM, Armonk, NY, USA) (training set: 999 patients; validation set: 684 patients).

As appropriate, the χ^2^-test, Student’s *t*-test, or Welch’s *t*-test was used to identify significant differences. Parameters with unequal variance were assessed with Welch’s *t*-test.

Repeated measures analysis of variance (RM ANOVA) with Bonferroni’s multiple comparisons was performed for intragroup analyses of changes in the FIB-4 index, serum albumin levels, PT activity (%), and AFP levels after DAA.

Factors that predicted HCC occurrence after DAA treatment were assessed with Cox proportional hazard model analyses. Significant predictors on univariate analysis were included in the multivariate analysis, and hazard ratios (HRs) and 95% confidence intervals (CIs) were calculated. Two-tailed *p* < 0.05 was considered significant. The Kaplan–Meier method was used to calculate the HCC incidence rate, and the log-rank test was used to calculate differences in the rates of new HCC. SPSS software ver. 23 was used for statistical analyses.

## Supplementary Information

Supplementary Information.

## Data Availability

The datasets generated during and/or analyzed during the current study are available from the corresponding author on reasonable request.

## Abbreviations

HCV: Hepatitis C virus
HCC: Hepatocellular carcinoma
DAAs: Direct-acting antivirals
SVR: Sustained virological response
IFN: Interferon
AFP: α-Fetoprotein
CT: Computed tomography
MRI: Magnetic resonance imaging
PT: Prothrombin activity
DM: Diabetes mellitus
EOT: End of treatment
HR: Hazard ratio
CI: Confidence interval
FIB-4: Fibrosis-4
AST: Aspartate aminotransferase
ALT: Alanine aminotransferase
