# Supplementary material for: Simple new clinical score to predict hepatocellular carcinoma after sustained viral response with direct-acting antivirals
Source: Sci Rep. 2023 Jun 2;13:8992. doi: 10.1038/s41598-023-36052-0 (PMC10238506; doi:10.1038/s41598-023-36052-0)
Supplement: Supplementary file 1 — Supplementary Information. [file 41598_2023_36052_MOESM1_ESM.pdf]

Simple new clinical score to predict hepatocellular carcinoma after sustained viral response with direct-acting antivirals

Takao Watanabe, Yoshio Tokumoto, Kouji Joko, Kojiro Michitaka, Norio Horiike, Yoshinori Tanaka, Atsushi Hiraoka, Fujimasa Tada, Hironori Ochi, Yoshiyasu Kisaka, Seiji Nakanishi, Sen Yagi, Kazuhiko Yamauchi, Makoto Higashino, Kana Hirooka, Makoto Morita, Yuki Okazaki, Atsushi Yukimoto, Masashi Hirooka, Masanori Abe, Yoichi Hiasa

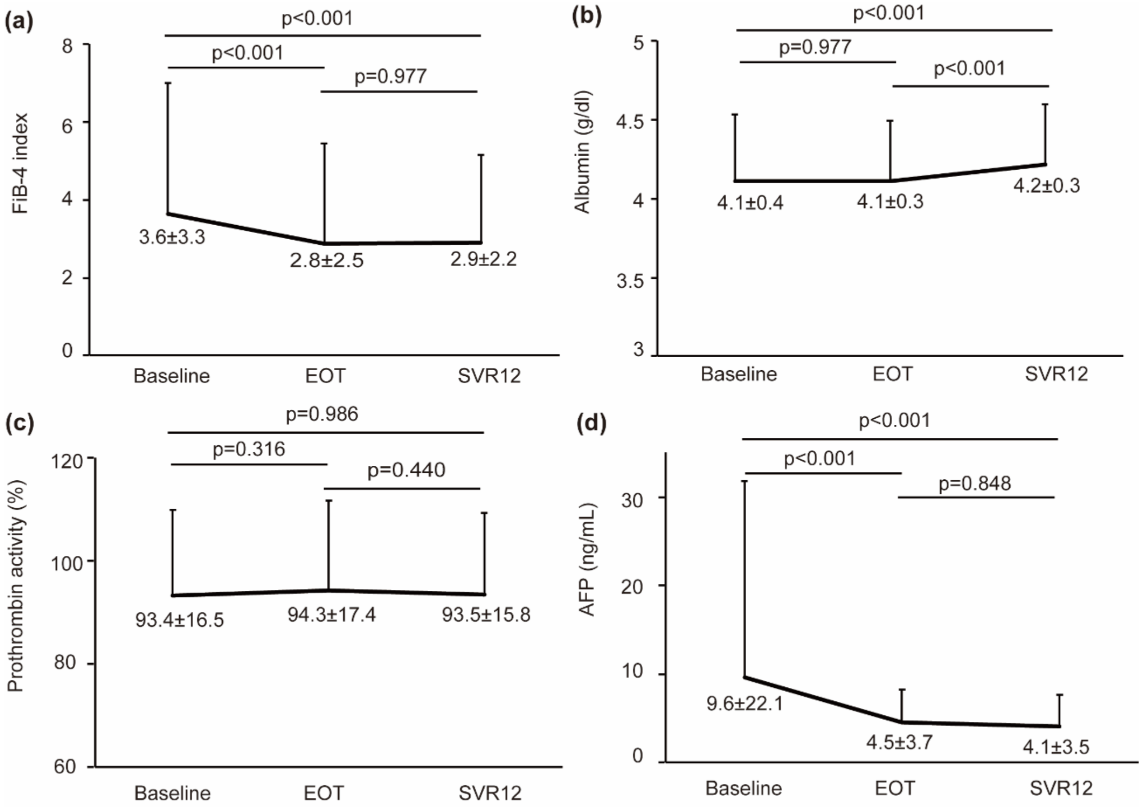

Figure S1.

FIB-4 index (a), serum albumin level (g/dL) (b), prothrombin activity (%) (c), and AFP (ng/ml) (d) values at baseline, EOT, and SVR12. The FIB-4 index is significantly lower at EOT and SVR12 than at baseline (a). P values were calculated with RM ANOVA with Bonferroni's multiple comparisons.

Table S1. Factors at treatment start including baseline factors associated with the occurrence of HCC after DAA treatment in the training set

	HCC occurrence		Univariate analysis		Multivariate analysis	
	Yes	No	Hazard ratio (95% CI)	p-value	Hazard ratio (95% CI)	p-value
Age (y)	68.9 ± 8.2	65.4 ± 10.3	1.03 (1.00-1.06)	0.029		
Male (n, %)	31, 67.3	416, 43.7	2.75 (1.48-5.10)	0.001	2.08 (1.06-40.4)	0.031
Body mass index (kg/m^2)	24.1 ± 3.1	23.2 ± 3.8	1.06 (0.98-1.14)	0.106		
Diabetes mellitus (n, %)	16, 34.7	154, 16.2	2.73 (1.49-5.02)	0.001	2.51 (1.27-4.95)	0.008
Alcohol (n, %)	5, 16.6	125, 16.7	1.17 (0.63-2.18)	0.60		
Smoking (n, %)	14, 6.3	32, 4.1	1.60 (0.85-3.00)	0.14		
HBV coinfection (n, %)	1, 8.3	45, 4.5	1.65 (0.22-12.0)	0.61		
White blood cell count (/µL)	4399 ± 1640	5108 ± 1630	0.99 (0.99-0.99)	0.009	0.99 (0.99-0.99)	0.001
Platelet count (×10^4/µL)	12.0 ± 5.7	16.6 ± 6.5	0.87 (0.83-0.92)	<0.001		
ALT (U/L)	51.5 ± 22.3	50.9 ± 46.5	1.00 (0.99-1.00)	0.89		

AST (U/L)	56.2 ± 29.2	48.4 ± 34.0	1.00 (0.99-1.01)	0.09		
LDL cholesterol (mg/dL)	93.3 ± 29.0	96.9 ± 28.9	0.99 (0.98-1.01)	0.52		
Triglycerides (mg/dL)	141 ± 56.6	116 ± 75.8	1.00 (0.99-1.00)	0.12		
Total bilirubin (mg/dL)	0.8 ± 0.3	0.8 ± 0.6	1.04 (0.69-1.57)	0.83		
Albumin level (g/dL)	3.9 ± 0.3	4.1 ± 0.4	0.35 (0.19-0.65)	<0.001		
Prothrombin activity (%)	83.0 ± 15.6	93.7 ± 16.9	0.97 (0.96-0.98)	<0.001	0.97 (0.96-0.99)	0.002
AFP level (ng/mL)	16.4 ± 17.9	9.9 ± 25.6	1.00 (0.99-1.01)	0.15		
FIB-4 index	4.8 ± 4.4	2.7 ± 2.0	1.04 (1.02-1.07)	<0.001	-	-

Data are expressed as means ± standard deviation.

HCC, hepatocellular carcinoma; DAA, direct-acting antiviral; CI, confidence interval; ALT, alanine aminotransferase; AST, aspartate aminotransferase; AFP, α-fetoprotein; FIB-4, fibrosis-4; SVR, sustained virological response

Table S2. Factors at the end of treatment associated with the occurrence of HCC after DAA treatment in the training set

	HCC occurrence		Univariate analysis		Multivariate analysis	
	Yes	No	Hazard ratio (95% CI)	p-value	Hazard ratio (95% CI)	p-value
Age (y)	68.9 ± 8.2	65.4 ± 10.3	1.03 (1.00-1.06)	0.029		
Male (n, %)	31, 67.3	416, 43.7	2.75 (1.48-5.10)	0.001	4.84 (1.83-12.8)	0.001
Body mass index (kg/m^2)	24.1 ± 3.1	23.2 ± 3.8	1.06 (0.98-1.14)	0.106		
Diabetes mellitus (n, %)	16, 34.7	154, 16.2	2.73 (1.49-5.02)	0.001	-	-
Alcohol (n, %)	5, 16.6	125, 16.7	1.17 (0.63-2.18)	0.60		
Smoking (n, %)	14, 6.3	32, 4.1	1.60 (0.85-3.00)	0.14		
HBV coinfection (n, %)	1, 8.3	45, 4.5	1.65 (0.22-12.0)	0.61		
White blood cell count (/μL)	4746 ± 1891	5249 ± 1753	1.00 (0.99-1.00)	0.11		
Platelet count (×10^4/μL)	13.3 ± 6.4	17.3± 7.0	0.90 (0.85-0.95)	<0.001		
ALT (U/L)	29.5 ± 35.3	20.0 ± 13.9	1.01 (1.00-1.01)	0.002		
AST (U/L)	33.5 ± 27.3	24.5 ± 11.3	1.01 (1.00-1.02)	<0.001		

Total bilirubin (mg/dL)	0.8 ± 0.4	0.8 ± 0.4	1.21 (0.70-2.08)	0.48		
Albumin level (g/dL)	4.0 ± 0.3	4.1 ± 0.3	0.41 (0.19-0.89)	0.024	-	-
Prothrombin activity (%)	84.4 ± 19.0	95.3 ± 17.0	0.97 (0.95-0.98)	<0.001	-	-
AFP level (ng/mL)	7.4 ± 5.5	4.2 ± 3.2	1.11 (1.06-1.17)	<0.001	1.13 (1.06-1.19)	<0.001
FIB-4 index	4.6 ± 4.5	2.7 ± 2.7	1.07 (1.03-1.11)	<0.001	1.09 (1.01-1.18)	0.028

Data are expressed as means ± standard deviation.

HCC, hepatocellular carcinoma; DAA, direct-acting antiviral; CI, confidence interval; ALT, alanine aminotransferase; AST, aspartate aminotransferase; AFP, α-fetoprotein; FIB-4, fibrosis-4; SVR, sustained virological response

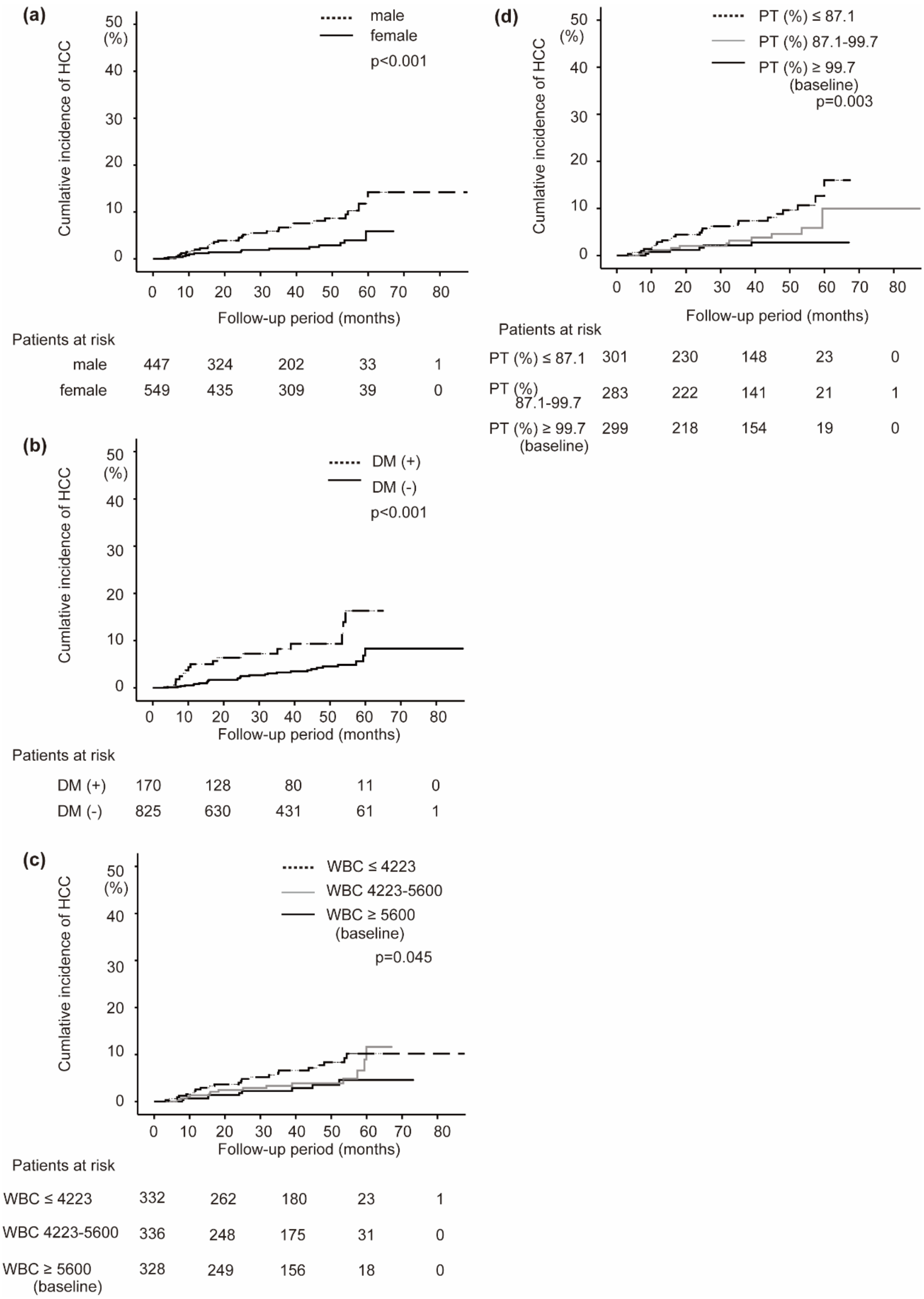

(a)
Cumlative incidence of HCC
50 (%)
40
30
20
10
0
0 10 20 30 40 50 60 70 80
Follow-up period (months)
male
female
p<0.001
Patients at risk
male 447 324 202 33 1
female 549 435 309 39 0
(b)
Cumlative incidence of HCC
DM (+)
DM (-)
p<0.001
Follow-up period (months)
Patients at risk
DM (+) 170 128 80 11 0
DM (-) 825 630 431 61 1
(c)
Cumlative incidence of HCC
WBC ≤ 4223
WBC 4223-5600
WBC ≥ 5600 (baseline)
p=0.045
Follow-up period (months)
Patients at risk
WBC ≤ 4223 332 262 180 23 1
WBC 4223-5600 336 248 175 31 0
WBC ≥ 5600 (baseline) 328 249 156 18 0
(d)
Cumlative incidence of HCC
PT (%) ≤ 87.1
PT (%) 87.1-99.7
PT (%) ≥ 99.7 (baseline)
p=0.003
Follow-up period (months)
Patients at risk
PT (%) ≤ 87.1 301 230 148 23 0
PT (%) 87.1-99.7 283 222 141 21 1
PT (%) ≥ 99.7 (baseline) 299 218 154 19 0

Figure S2.

Comparison of the cumulative HCC occurrence rate in the training set. The cumulative occurrence of HCC is significantly stratified by sex ($p < 0.001$) (a), DM ($p < 0.001$) (b), white blood cell count (/μL) ($p=0.045$) (c), and prothrombin activity (%) ($p = 0.003$) (d) with the log-rank test.

(a)

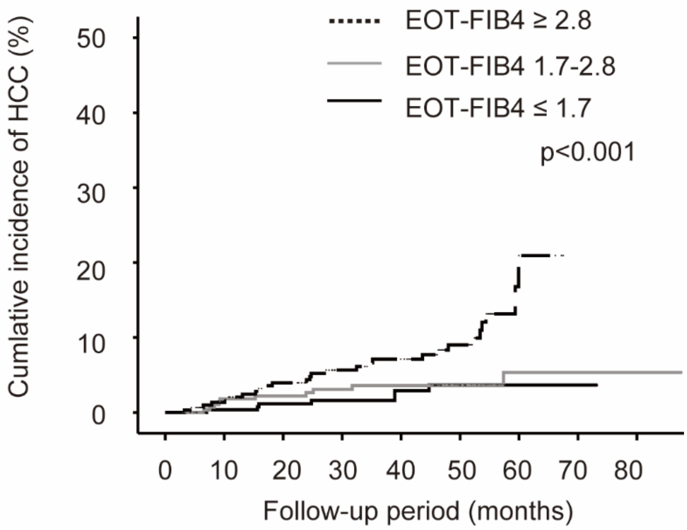

Patients at risk

EOT-FIB4 ≥ 2.8	312	245	172	18	0
EOT-FIB4 1.7-2.8	307	236	161	26	1
EOT-FIB4 ≤ 1.7	293	234	149	23	0

(b)

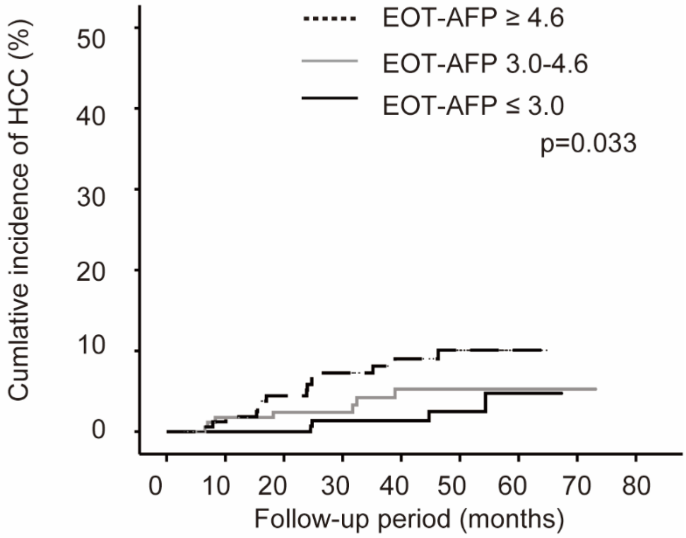

Patients at risk

EOT-AFP ≥ 4.6	183	137	98	11
EOT-AFP 3.0-4.6	188	145	88	13
EOT-AFP ≤ 3.0	185	152	106	11

Figure S3.

Comparison of the cumulative HCC occurrence rate in the training set. There was a significant difference among the patients by the FIB-4 index at EOT ($p < 0.001$) (a), and by the AFP level (ng/mL) at EOT ($p = 0.033$) (b) with the log-rank test.

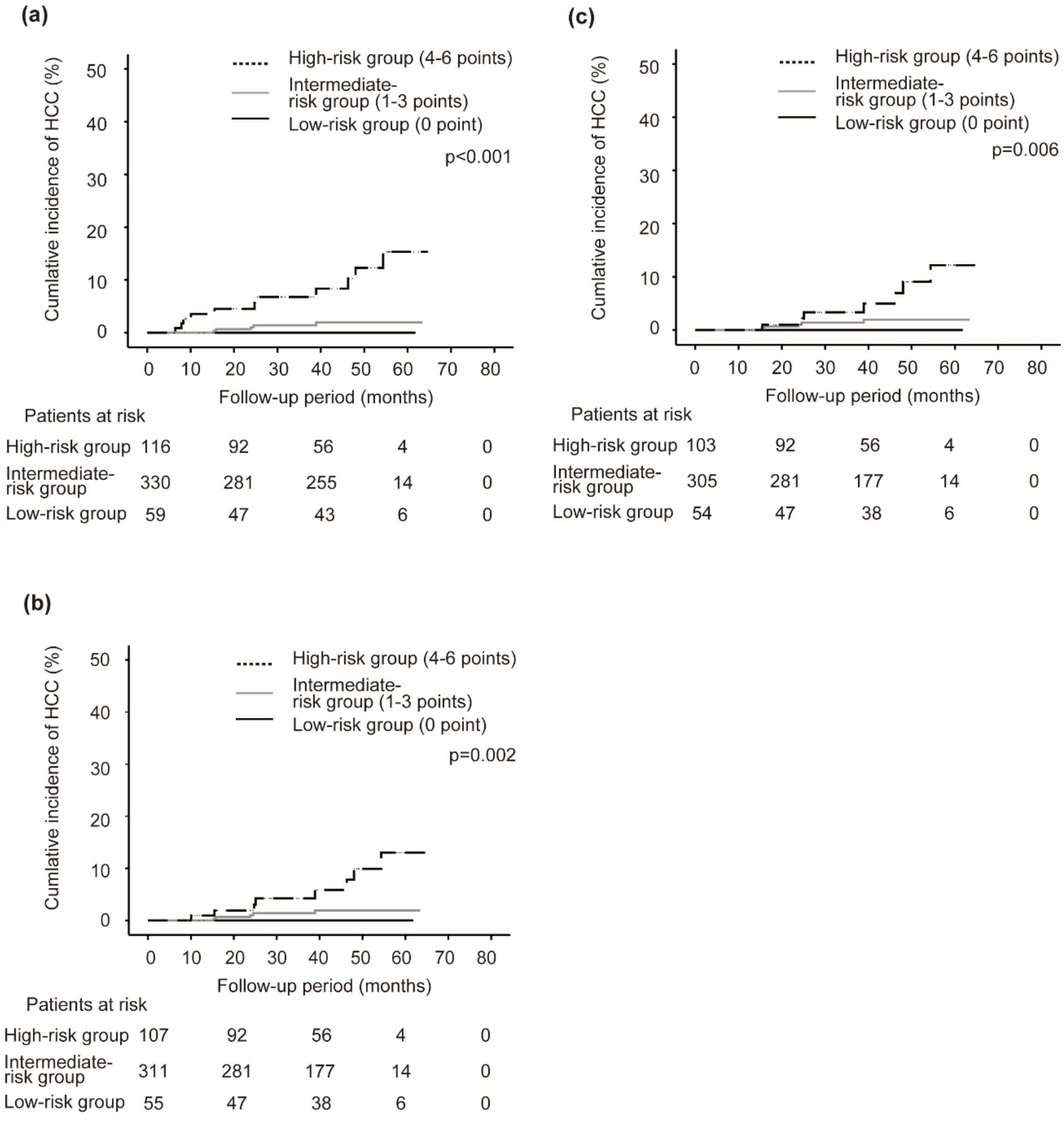

Figure S4.

Comparison of the cumulative HCC incidence in the training set by the scoring systems with factors at SVR12 excluding patients who developed HCC within 6, 9, and 12 months after the end of DAA treatment. The cumulative occurrence of HCC by group is significantly stratified when excluding patients who developed HCC within 6 ($p < 0.001$) (a), 9 ($p = 0.002$) (b), and 12 months ($p = 0.006$) (c) with the log-rank test.
